# GALNT14 deficiency: connecting multiple links in the IgA nephropathy pathogenetic chain

**DOI:** 10.1172/JCI192687

**Published:** 2025-05-15

**Authors:** John Pell, Madhav C. Menon

**Affiliations:** Division of Nephrology, Department of Medicine & Human Translational Immunology program, Yale University School of Medicine, New Haven, Connecticut, UDA.

## Abstract

IgA nephropathy (IgAN) is a highly prevalent type of primary glomerulonephritis. IgAN involves mesangial deposition of immune complexes leading to complement activation, inflammation, and glomerular injury. A key hit for pathogenesis involves aberrant O-glycosylation in the hinge region of IgA. Despite its prevalence, however, the mechanisms underlying IgAN remain incompletely understood. In this issue of the *JCI*, Prakash and colleagues used whole-exome sequencing of two IgAN probands to identify loss-of-function variants in *GALNT14* leading to loss of the enzyme GalNAc-T14, which is involved in O-glycosylation. The authors then performed a classical bedside-to-bench investigation using a *Galnt14^–/–^* mouse model and connected loss of GalNAc-T14 to excess IgA production, impaired B lymphocyte homing, and defective intestinal mucus production. These findings build a more unified understanding of IgAN pathogenesis from defective O-glycosylation with loss-of-function variants in *GALNT14*.

## IgAN risk

Globally, IgA nephropathy (IgAN) is a leading form of primary glomerulonephritis. Disease incidence is racially stratified, with a particularly high frequency among East Asian and Western European populations (1). As many as 50% of patients with IgAN will progress to end-stage kidney disease (ESKD) within their lifetime (2, 3), highlighting the need for targeted therapeutics.

Sequentially, several events in disease progression have been identified in IgAN (reviewed in ref. 4–8). An initiating event involves production of IgA1 with aberrant O-glycosylation in the hinge region reducing galactosylation (Gd-IgA1), followed by production of anti-Gd-IgA1 antibodies and subsequent formation of Gd-IgA1/anti-Gd-IgA1 immune complexes. These complexes deposit in the mesangial region, leading to complement activation, mesangial cell proliferation, recruitment of inflammatory cells, and podocyte injury, all of which drive glomerular destabilization and progressive CKD (9). Defective B lymphocyte homing from mucosal sites to lymphoid organs has been suggested as an important contributor to pathogenesis (10). Indeed, recent GWAS have demonstrated that IgAN risk loci localize to multiple genes related to immune modulatory traits and support a polygenic architecture for IgAN (11). Despite these insights, in any given case, pathomechanisms that convert risk genotypes to IgAN phenotype remain incompletely understood. For instance, the specific role played by respiratory or gastrointestinal infections in inducing or aggravating each case of IgAN is not entirely clear.

## GalNAc-T14 loss impairs B cell homing through O-glycosylation defects

The study by Prakash et al. (12) published in this issue of the *JCI* takes a bedside-to-bench approach, first localizing likely pathogenic mutations in patients with IgAN and then mechanistically investigating these mutations using a *Galnt14-*null mouse model. The authors identified a family in Italy with a history of IgAN consistent with autosomal dominant inheritance. Genome-wide genotyping of the family supplemented by whole exome sequencing of two separate probands — one with biopsy-proven recurrent IgAN and his mother, who presented with microscopic hematuria — pinpointed a loss-of-function (LOF) mutation in *GALNT14*, which was the only predicted LOF variant within linkage intervals and present in all affected individuals. To conclusively demonstrate an association between *GALNT14* LOF variants with exome-wide significance, a cohort of 4,000–10,000 IgAN cases would be required; however, this variant was present in approximately 1-in-500 individuals with IgAN and had an approximate 5-fold higher frequency among individuals with IgAN. Moreover, overexpression of *GALNT14* — which encodes the enzyme GalNAc-T14 — has been previously shown to increase galactose-deficient IgA1 (Gd-IgA1) production in vitro (13), supporting a role for this mutation in IgAN pathology.

Interestingly, Prakash and authors found that *GALNT14* mRNA localized to the germinal centers (GC) of both human and murine spleen and lymph nodes, which are major sites for B cell maturation. They also identified GalNAc-T14 expression as highest in proximal and distal tubules of the nephron, potentially pointing to a role for O-glycosylation of epithelial surface mucins. To investigate the consequences of loss of GalNAc-T14, the authors utilized a mouse model with a germline inactivation of *Galnt14* (*Galnt14^–/–^* mice). The authors then conducted a detailed comparison of immunological and histological characteristics between *Galnt14^–/–^* mice and *Galnt14^+/–^* as well as *Galnt14^+/+^* controls. Null mice had no obvious abnormalities in fertility or gross histology of major organs. However, they showed elevated levels of serum IgA, and alcian blue staining revealed a reduced mucin layer in the colons of 8-week-old adults. In homeostasis, young *Galnt14^–/–^* mice did not develop any overt glomerular pathology or IgA deposition. However, aging or induction of chemical sterile colitis resulted in mesangial IgA deposition. Additionally, *Galnt14^–/–^* mice showed elevated IgA production from B cells at multiple mucosal sites (including IgA bound to bacteria in the small and large intestines). Critically, J-Chain–containing IgA (representing polymeric IgA) was elevated in *Galnt14^–/–^* sera. Spleen-derived *Galnt14^–/–^* B cells showed both elevated IgA and IgG concentrations in supernatants versus *Galnt14^+/+^* B cells. Simultaneously, 16S RNA analysis of the gut microbiome showed that gut microbiome alterations did not play a role in determining IgA or IgG levels or deposition in *Galnt14^–/–^* mice.

Next, the authors used flow cytometry to identify surface-IgA^+^ B cells in immune tissues, revealing altered distribution of IgA^+^ B cells in *Galnt14^–/–^* mice — with increases in PBMCs, spleen, and the peritoneal cavity, but decreases in the Peyer’s Patches, suggesting a redistribution between lymphoid tissue and mucosal surfaces. They hypothesized that *Galnt14* deficiency would inhibit the O-glycosylation of surface glycoproteins in B cells and correspondingly showed reduced peanut agglutinin (PNA) lectin staining in Splenic and Peyer’s patch GC B cells. The number of overall GC B cells, and, moreover, the number and proportion of PNA^+^ B cells, were specifically reduced in Peyer’s patch GCs. The authors concluded that reduced O-glycosylation of surface glycoproteins in *Galnt14^–/–^* GC B cells likely leads to inhibited ability to home and maintain residence in GCs, especially within Peyer’s patches. Finally, adoptive transfer experiments demonstrated this fundamental intrinsic homing defect in *Galnt14^–/–^* B cells. This homing defect was specific to B cells with *Galnt14* deficiency and spared T cell homing abilities, possibly because of low GalNAc-T14 expression in T cells at baseline.

## Conclusion and implications

**Figure 1:**
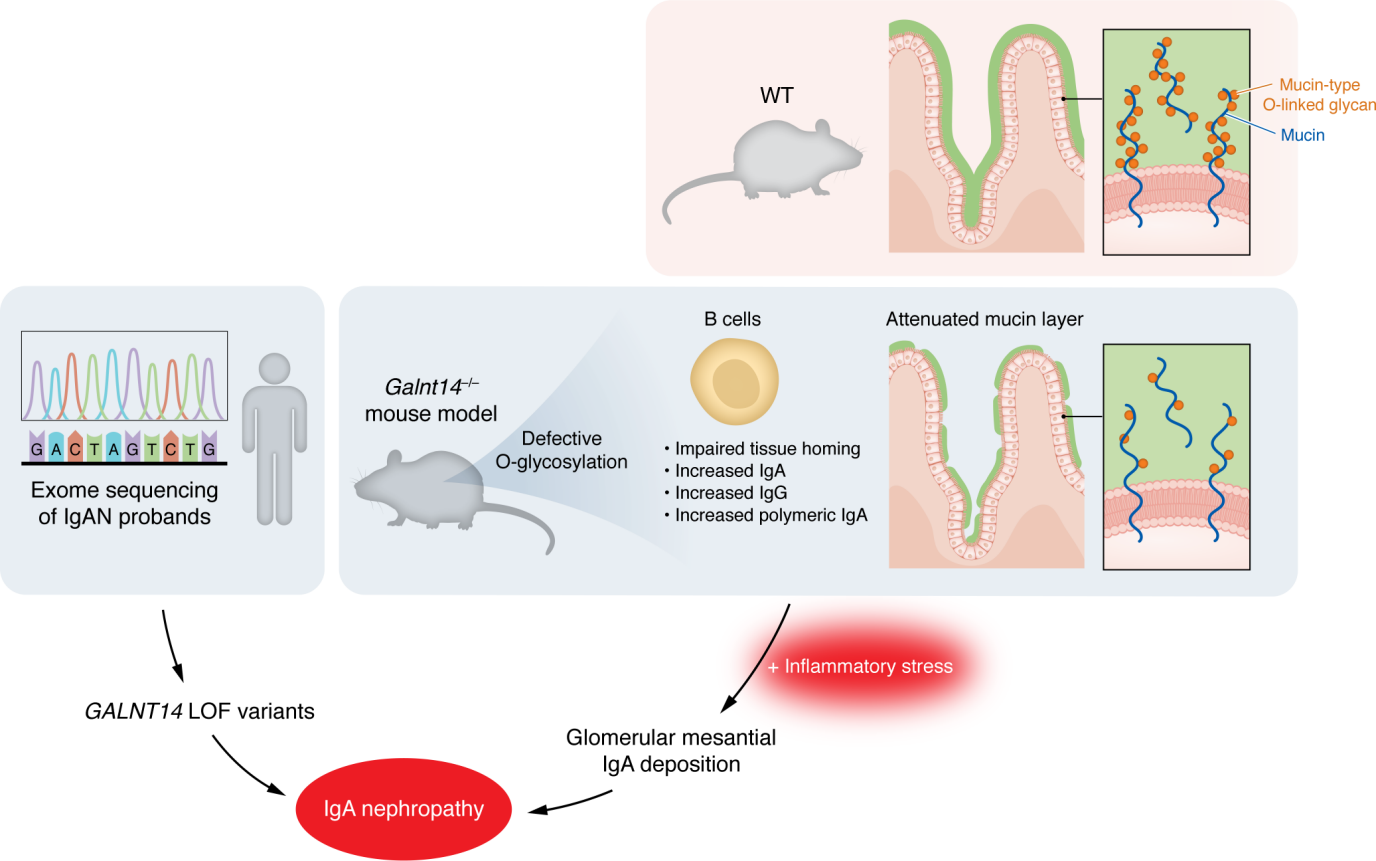
Loss of GalNAc-T14 connects IgAN-related phenotypes. Whole exome sequencing of two IgAN probands revealed LOF mutations in GALNT14, a gene that encodes the galactosyl transferase GalNAc-T14 involved in O-glycosylation. GalNAc-T14 is known to participate in mucin-type O-glycosylation and Galnt14^–/–^ mice showed an attenuated mucin layer as well as altered B lymphocyte homing and elevated IgA and IgG production. In response to stressors such as aging or sterile colitis, these mice developed glomerular mesangial IgA deposition.

Overall, this comprehensive series of experiments by Prakash et al. (12) connects multiple links in the IgAN pathogenetic chain to a single genetic defect in *GALNT14*. First, *Galnt14* deficiency in mice caused excess total IgA, and corresponding *GALNT14*-LOF variants in humans also tended to increase total serum IgA in the patient cohort. Specifically, polymeric-IgA levels, important in IgAN pathogenesis (14), were also increased in sera of *Galnt14^–/–^ mice*. Next, the observed increases in IgA along with IgG allow for the concept that *Galnt14* deficiency could, in addition to increased IgA, enhance IgA-IgG complex formation important in IgAN pathogenesis. The use of a mouse model allowed the authors to specifically study non Gd-IgA1 effects of GalNAc-T14–deficiency, since murine IgA lacks the O-glycosylated hinge region of human IgA. The global defect with O-glycosylation seen in *Galnt14^–/–^* reduced the intestinal mucin layer and induced glomerular IgA deposition after intestinal inflammatory events, which are also known to precede IgAN exacerbations in humans. This finding (though not specifically tested in the human probands in the study) could explain a well-known “gut-to-glomerulus” link for IgAN observed in inflammatory bowel diseases and celiac enteropathy (15). However, whether patients with inflammatory bowel disease and/or celiac disease who develop IgAN show specific enrichments of *GALNT14* mutations or LOF variants needs further investigation. The defective O-glycosylation of multiple surface glycoproteins observed in *Galnt14^–/–^* B cells, which likely induces the defective tissue homing, however, did not translate to altered Gd-IgA1 levels in the human cohort, suggesting redundancy of O-glycosylation mechanisms (16). While IgAN-related B cell homing defects were identified by Prakash et al. (12) and elsewhere (10), the relative sparing of T cell homing with this defect would coincidentally allow T cell–mediated glomerular injury to continue, contributing to glomerulonephritis in IgAN. From a clinical perspective, although the mouse data are comprehensive, it must be noted that these mutations are rare in the human population and were not observed in previous IgAN GWAS (11). Importantly, the *GALNT14-*LOF variants follow an autosomal dominant inheritance, albeit with incomplete penetrance, similar to the heritability of familial IgAN observed in large cohorts (17, 18). The high phenotypic impact of this monogenic variant could ultimately be explained by its simultaneous intersection with multiple pathogenetic steps in IgAN (Figure 1). In summary, Prakash et al. (12) is an exciting study that exemplifies patient-based, focused genetic studies. The rigorous in vitro and in vivo experiments shed new light on the polygenic and mechanistic architecture of IgA nephropathy.

## Version 1 05/15/2025

Electronic publication
